# Supplementary material for: DEPs Induce Local Ige Class Switching Independent of Their Ability to Stimulate iBALT de Novo Formation
Source: Int J Environ Res Public Health. 2022 Oct 11;19(20):13063. doi: 10.3390/ijerph192013063 (PMC9603618; doi:10.3390/ijerph192013063)
Supplement: Supplementary file 1 [file ijerph-19-13063-s001.zip › Supplementary materials 10-2022/Supplementary Table S1.pdf]

Supplementary Table S1. Primers and probed used in this study

Gene/ transcript	Primer/ Probe	Sequence
GAPDH	F	GGAGAGTGTTTCCTCGTCCC
	R	ACTGTGCCGTTGAATTTGCC
	Z	/6-FAM/-CGCCTGGTCACCAGGGCTGCCATTTGCAGT-/BHQ-1/
HPRT	F	CAGTCCCAGCGTCGTGATTA
	R	TCCAGCAGGTCAGCAAAGAA
	Z	/6-FAM/-TGGGAGGCCATCACATTGTGGCCCTCTGTGTG-/BHQ-1/
germline ε	F	CCCACTTTTAGCTGAGGGCA
	R	CTGGTTAAGGGCAGCTGTGA
	Z	/6-FAM/-CGCCTGGGAGCCTGCACAGGGGGC-/BHQ-1/
germline γ1	F	AGAACCAAGGAAGCTGAGCC
	R	AGTTTGGGCAGCAGATCCAG
	Z	/6-FAM/-AGGGGAGTGGGCGGGGAGGCCA-/BHQ-1/
postswitch ε	F	CCAGTCCACATGCTCTGTGT
	R	AGCGTGGGGAACTGGTTAAG
	Z	/6-FAM/-TGGGGTCCCCAGAGCCCTGCTCCTGT-/BHQ-1/
postswitch γ1	F	CCTCTGGCCCTGCTTATTGT
	R	GTCACTGTCACTGGCTCAGG
	Z	/6-FAM/-CCACTGGCCCCTGGATCTGCTGCCCA-/BHQ-1/
circular μ-ε	F	CCCACTTTTAGCTGAGGGCA
	R	CGAGGGGGAAGACATTTGGG
	Z	/6-FAM/-CGCCTGGGAGCCTGCACAGGGGGC-/BHQ-1/
circular γ1-ε	F	AGATTCACAACGCCTGGGAG
	R	GTCACTGTCACTGGCTCAGG
	Z	/6-FAM/-CCACTGGCCCCTGGATCTGCTGCCCA-/BHQ-1/
Tnfa	F	TCTTCTCAAAATTCGAGTGACAAGC
	R	GATAGCAAATCGGCTGACGGT
	Z	/6-FAM/-AGGCTGCCCCGACTACGTGCTCCTCACCCA-/BHQ-1/
Ifna1	F	TGCCCAGCAGATCAAGAAGG
	R	TCAGGGGAAATTCCTGCACC
	Z	/6-FAM/-CCTGCAAGGCTGTCTGATGCAGCAGGTGGG-/BHQ-1/
Il4	F	CCATATCCACGGATGCGACA
	R	AAGCACCTTGGAAGCCCTAC
	Z	/6-FAM/-AGGGACGCCATGCACGGAGATGGATGTGCC-/BHQ-1/
Il13	F	GTGTCTCTCCCTCTGACCCT
	R	TCTGGGTCCTGTAGATGGCA
	Z	/6-FAM/-CCGCTGGCGGGTTCTGTGTAGCCCTGGATT-/BHQ-1/
Ifng	F	TCAGGCCATCAGCAACAACA
	R	CTTCCTGAGGCTGGATTCCG
	Z	/6-FAM/-AGGTCAACAACCCACAGGTCCAGCGCCAAG-/BHQ-1/
Cd19	F	GAGGCACGTGAAGGTCATTG
	R	TTGAAGAATCTCCTGGCGGG
	Z	/6-FAM/-AGCAGTGTGGCTCTGGCTGTTGAGAACTGGTG-/BHQ-1/
Bcl6	F	CACTATAGGGCGGCGAGC
	R	TGCCTTGCTTCACAGTCCAA
	Z	/6-FAM/-CCCCTGCTGCGGAGCAATGGTAAAGCCCGC-/BHQ-1/
Ebi2	F	CAAACACGGACTGCCACAAC
	R	CAATGACAACCAAGGCCAGC
	Z	/6-FAM/-TCTCTATGCCCACCACAGCACAGCCAGGGT-/BHQ-1/
