# Supplementary figures and images for: DEPs Induce Local Ige Class Switching Independent of Their Ability to Stimulate iBALT de Novo Formation
Source: Int J Environ Res Public Health. 2022 Oct 11;19(20):13063. doi: 10.3390/ijerph192013063 (PMC9603618; doi:10.3390/ijerph192013063)

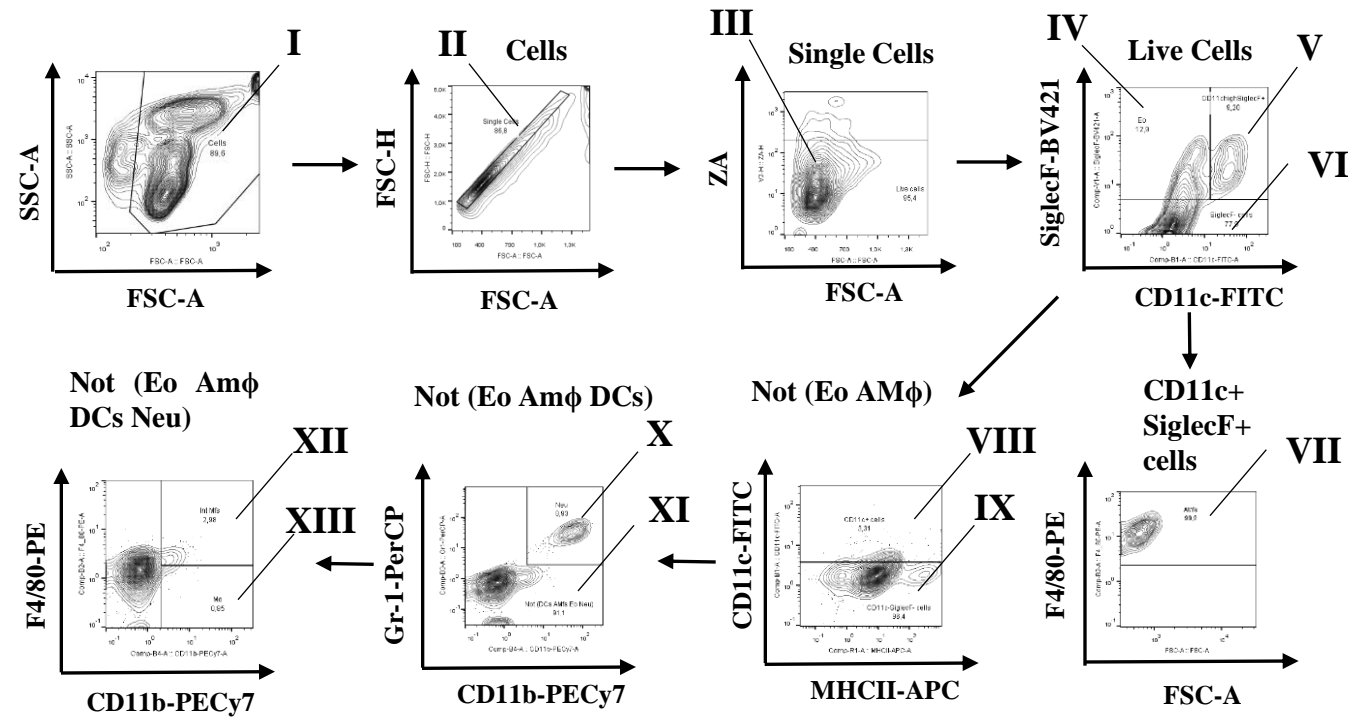

**Figure S1**

Supplement: Supplementary file 1 [file ijerph-19-13063-s001.zip › Supplementary materials 10-2022/Figure S1 09-09-22.pdf]

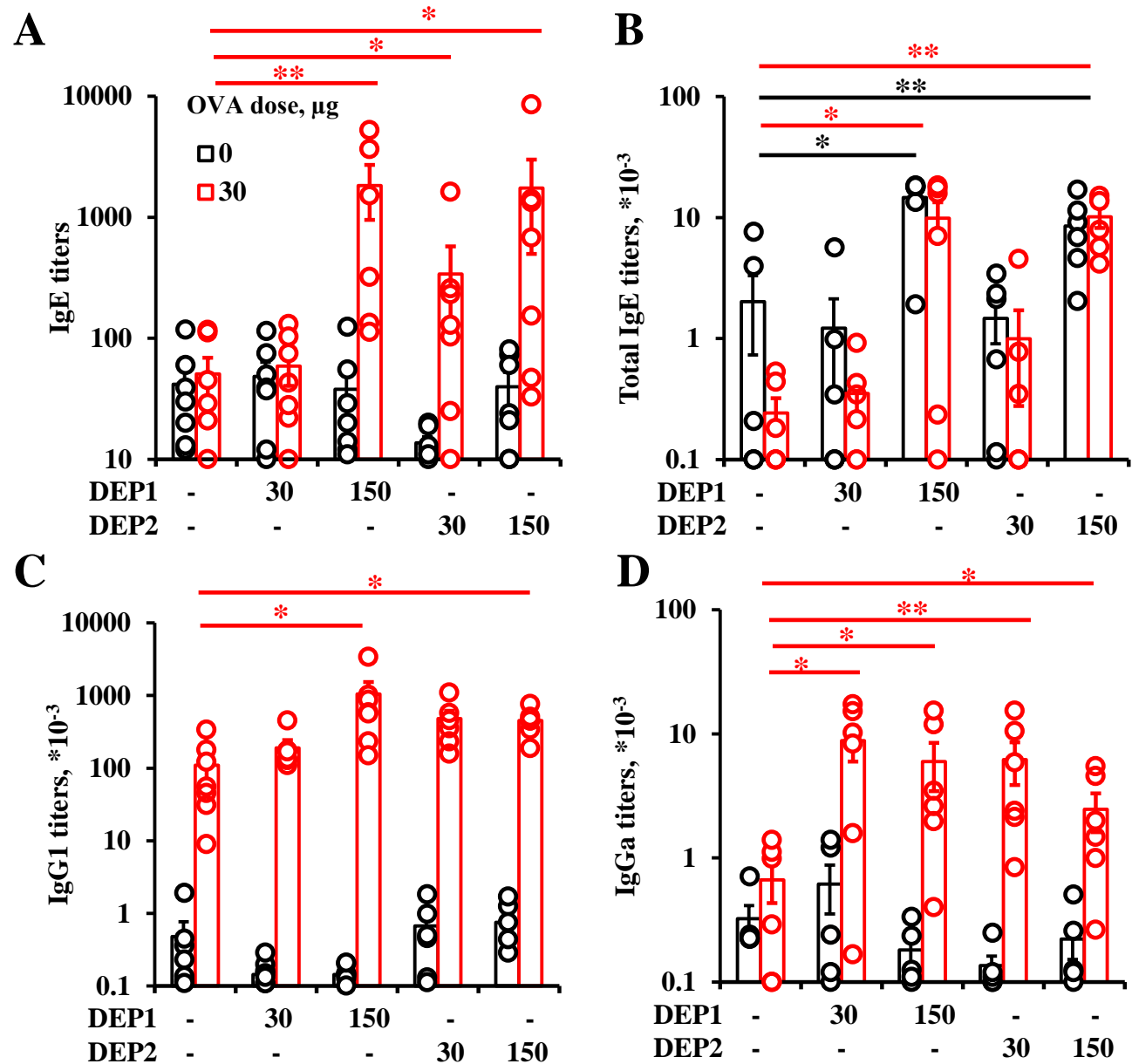

Figure S2

Supplement: Supplementary file 1 [file ijerph-19-13063-s001.zip › Supplementary materials 10-2022/Figure S2 09-09-22.pdf]

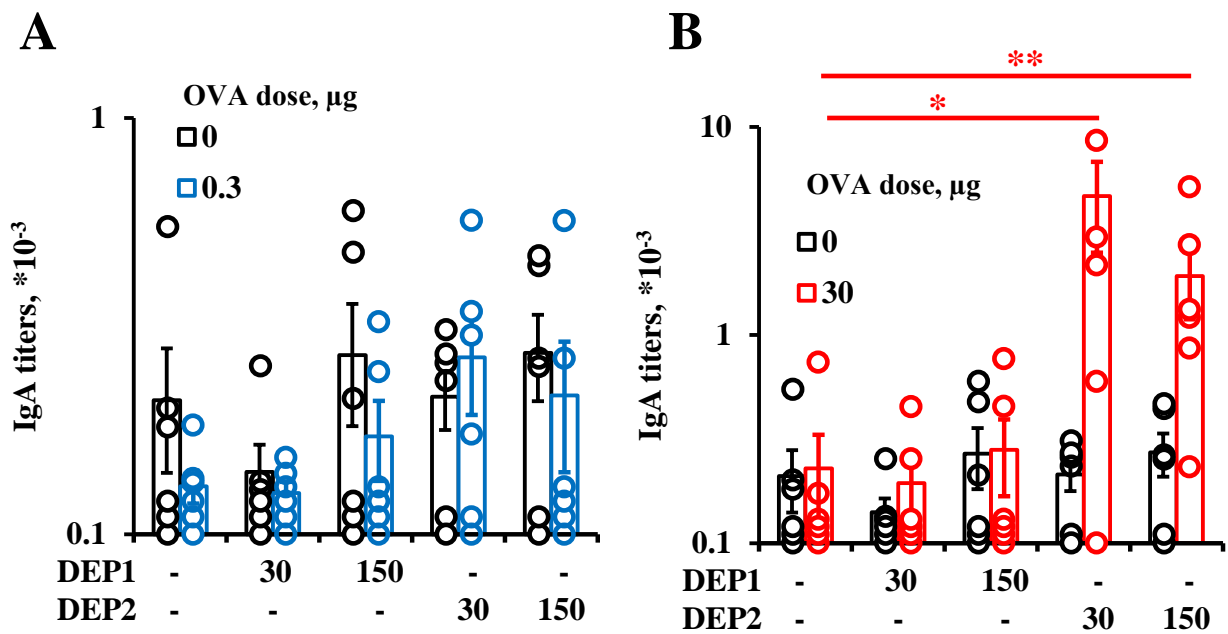

Supplement: Supplementary file 1 [file ijerph-19-13063-s001.zip › Supplementary materials 10-2022/Figure S3 09-09-22.pdf]

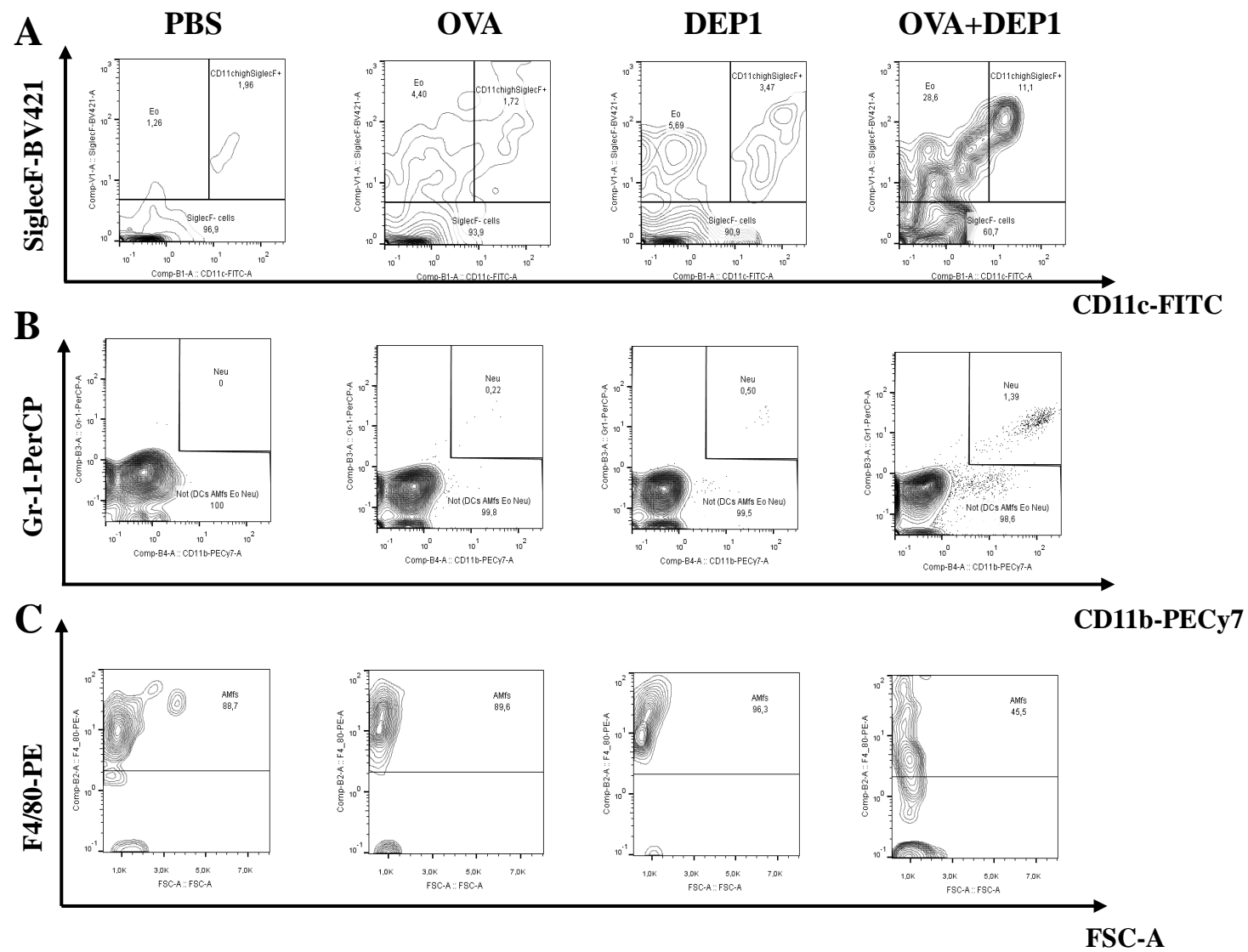

**Figure S4**

Supplement: Supplementary file 1 [file ijerph-19-13063-s001.zip › Supplementary materials 10-2022/Figure S4 09-09-22.pdf]

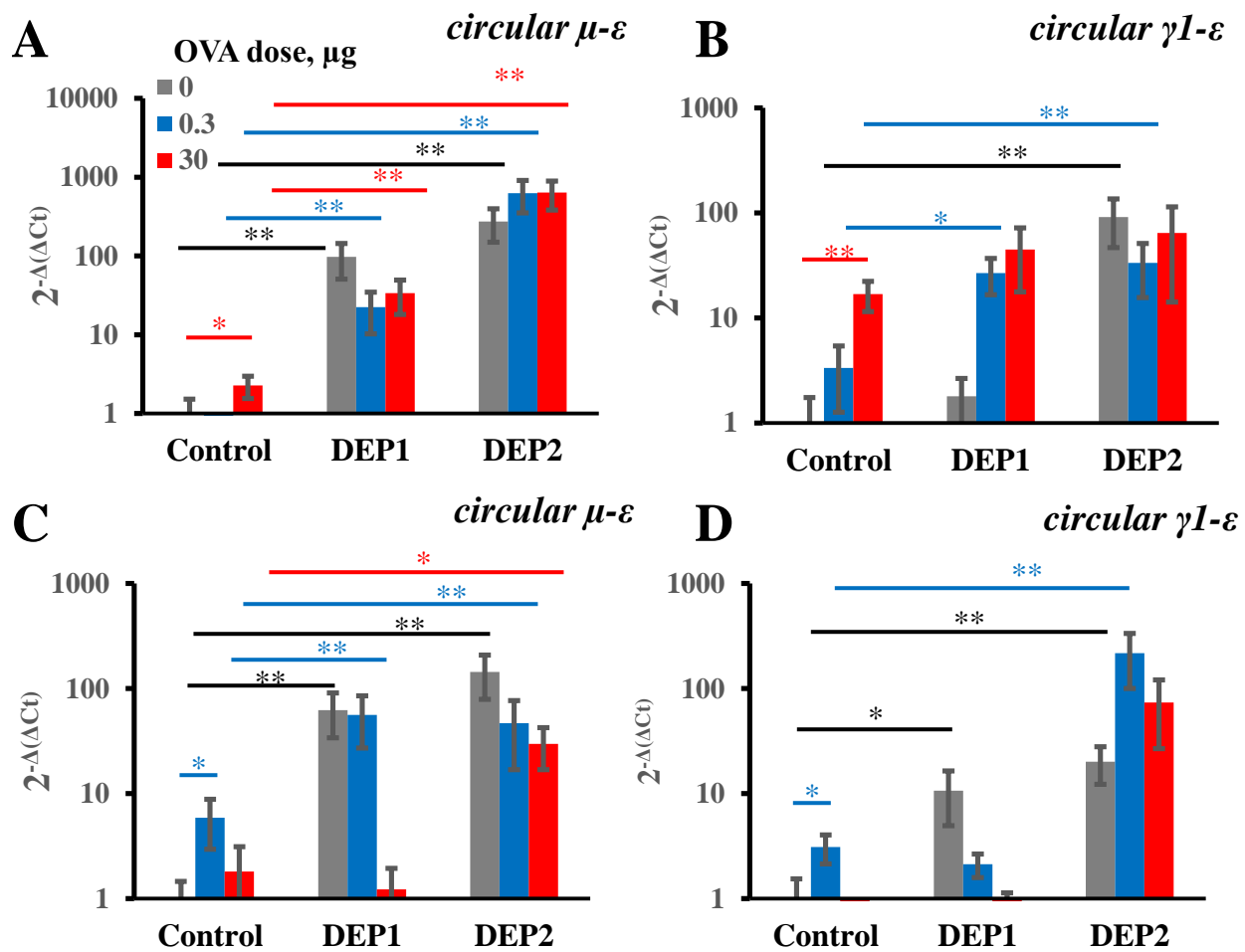

**Figure S5**

Supplement: Supplementary file 1 [file ijerph-19-13063-s001.zip › Supplementary materials 10-2022/Figure S5 09-09-22.pdf]

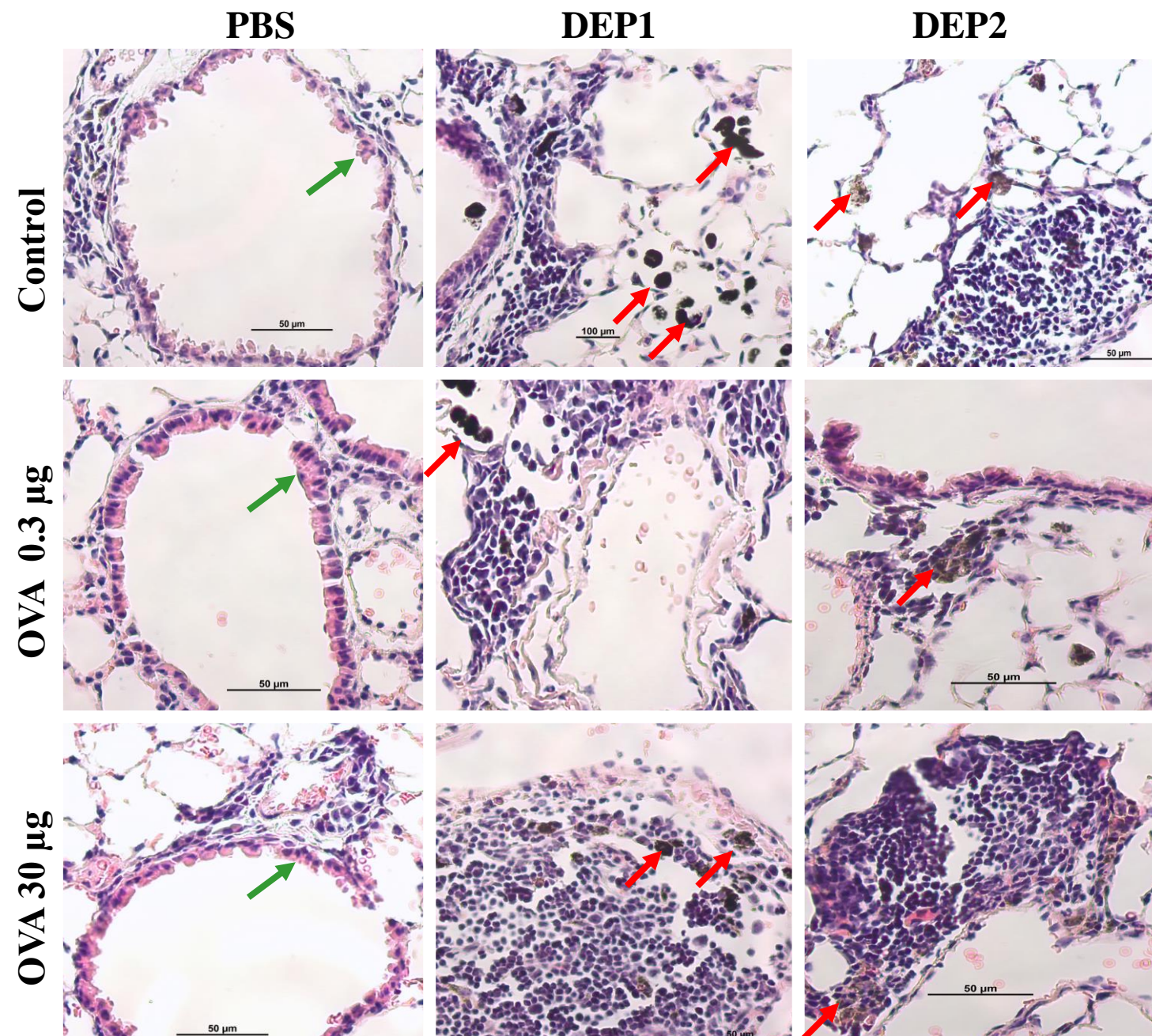

**Figure S6**

Supplement: Supplementary file 1 [file ijerph-19-13063-s001.zip › Supplementary materials 10-2022/Figure S6 09-09-22.pdf]
